# The tyrosine kinase receptor Tyro3 enhances lifespan and neuropeptide Y (Npy) neuron survival in the mouse anorexia (*anx*) mutation

**DOI:** 10.1242/dmm.027433

**Published:** 2017-05-01

**Authors:** Dennis Y. Kim, Joanna Yu, Ryan K. Mui, Rieko Niibori, Hamza Bin Taufique, Rukhsana Aslam, John W. Semple, Sabine P. Cordes

**Affiliations:** 1 Lunenfeld-Tanenbaum Research Institute, Room 876, Mount Sinai Hospital, 600 University Avenue, Toronto, ON M5G 1X5, Canada; 2 Department of Molecular Genetics, University of Toronto, 1 King's Crescent, Toronto, ON M5S 1A8, Canada; 3 Keenan Research Centre for Biomedical Science, St. Michaels Hospital, Toronto, ON M5B 1W8, Canada; 4 Canadian Blood Services, 67 College Street, Toronto, ON M5G 2M1, Canada; 5 Department of Laboratory Medicine and Pathobiology, University of Toronto, Toronto, ON M5S 1A1, Canada

**Keywords:** Eating disorder, RNA localization, Appetite regulation, Neurodegeneration, Npy, Modifier, Transgenic rescue

## Abstract

Severe appetite and weight loss define the eating disorder anorexia nervosa, and can also accompany the progression of some neurodegenerative disorders such as amyotrophic lateral sclerosis (ALS). Although acute loss of hypothalamic neurons that produce appetite-stimulating neuropeptide Y (Npy) and agouti-related peptide (Agrp) in adult mice or in mice homozygous for the anorexia (*anx*) mutation causes aphagia, our understanding of the factors that help maintain appetite regulatory circuitry is limited. Here we identify a mutation (C19T) that converts an arginine to a tryptophan (R7W) in the TYRO3 protein tyrosine kinase 3 (*Tyro3*) gene, which resides within the *anx* critical interval, as contributing to the severity of *anx* phenotypes. Our observation that, like *Tyro3^−/−^* mice, *anx/anx* mice exhibit abnormal secondary platelet aggregation suggested that the C19T *Tyro3* variant might have functional consequences. *Tyro3* is expressed in the hypothalamus and other brain regions affected by the *anx* mutation, and its mRNA localization appeared abnormal in *anx/anx* brains by postnatal day 19 (P19). The presence of wild-type *Tyro3* transgenes, but not an *R7W-Tyro3* transgene, doubled the weight and lifespans of *anx/anx* mice and near-normal numbers of hypothalamic Npy-expressing neurons were present in *Tyro3*-transgenic *anx/anx* mice at P19. Although no differences in R7W-Tyro3 signal sequence function or protein localization were discernible *in vitro*, distribution of R7W-Tyro3 protein differed from that of Tyro3 protein in the cerebellum of transgenic wild-type mice. Thus, R7W-Tyro3 protein localization deficits are only detectable *in vivo*. Further analyses revealed that the C19T *Tyro3* mutation is present in a few other mouse strains, and hence is not the causative *anx* mutation, but rather an *anx* modifier. Our work shows that Tyro3 has prosurvival roles in the appetite regulatory circuitry and could also provide useful insights towards the development of interventions targeting detrimental weight loss.

## Abstract (precis)

**Summary:** The receptor tyrosine kinase *Tyro3* improves weight and survival in the anorexia mouse model, and is one of the few factors known to sustain appetite regulatory circuitry.

## INTRODUCTION

Detrimental appetite and weight loss are the defining features of the restrictive subtype of the eating disorder anorexia nervosa (ANR) (Kaye et al., 1998, 2000; Walsh and Devlin, 1998), and also often accompany a range of neurodegenerative disorders, including amyotrophic lateral sclerosis (ALS) (Ahmed et al., 2016), Alzheimer’s disease (AD) (Ringman et al., 2015; Burke et al., 2016; Sergi et al., 2013) and Huntington's disease (HD) (Petersén et al., 2005; Gabery et al., 2010). Eating disorders have the highest mortality rate of any mental illness (Sullivan, 1995). Moreover, appetite and weight loss are linked to poor mood and quality of life in ALS patients and can be major issues for many during late disease progression. In spite of their impact, the neurobiological systems (Kaye et al., 2013) and genetic factors (Shih and Woodside, 2016; Clarke et al., 2012; Pinheiro et al., 2009) that contribute to reduced food intake in ANR remain poorly understood and are likely complex, and have been largely unexplored in neurodegenerative disorders.

Neuronal circuitry that links the hypothalamus with extra-hypothalamic regions, including the parabrachial nucleus and the nucleus accumbens, monitors peripheral energy levels and regulates food intake (reviewed in Sternson, 2013; Morton et al., 2006). Within the hypothalamus, orexigenic neurons co-express and release neuropeptide Y (Npy) (Allen et al., 1983; Chronwall et al., 1985; de Quidt and Emson, 1986; Hahn et al., 1998) and agouti-related protein (AgRP) (Hahn et al., 1998; Shutter et al., 1997) to promote feeding by inhibiting anorexic neurons expressing pro-opiomelanocortin (Pomc) (Bloch et al., 1978; Watson et al., 1978) and cocaine- and amphetamine-regulated transcript (Cart, also known as Cartpt) (Elias et al., 1998; Kristensen et al., 1998). Serotonin can act directly on 5HT2c receptors present in hypothalamic orexigenic neurons and 5HT1b receptors in anorexigenic neurons (Garfield and Heisler, 2009; Heisler et al., 2002, 2006; Lam et al., 2008; Xu et al., 2008). In the adult mouse, acute ablation of arcuate nucleus AgRP neurons leads to starvation (Luquet et al., 2005; Xu et al., 2005; Wu et al., 2008). AgRP neurons also project long-range axons to other brain regions, including the parabrachial nucleus (PBN), which can augment or suppress AgRP neuron actions. Further analyses in the mouse have shown that the severe aphagia caused by acute adult AgRP neuron loss can be reversed by eliminating glutamatergic excitatory drive or increasing GABAergic inhibition from the PBN or by inhibiting serotonergic activation of the nucleus of the solitary tract, which innervates and activates the PBN (Wu et al., 2012). Thus, in brief, loss of hypothalamic NPY/AGRP neurons, increased glutamatergic signaling from the PBN or serotonergic hyperinnervation of the nucleus tractus solitarius (NTS) could individually or together promote pathological appetite and weight loss.

Understanding how appetite regulatory circuits are involved or have been compromised in individual ANR cases, let alone in ANR populations, is largely unknown. Because prior to their diagnosis ANR patients have already been undernourished for an extended period of time, it is difficult to distinguish between the neurobiological causes and effects of ANR. Furthermore, imaging deeper brain regions such as the hypothalamus can be more challenging (Val-Laillet et al., 2015). Nonetheless, data concerning appetite regulating peptide levels in ANR patients have been obtained by assessing cerebrospinal fluid and plasma levels, and persistent changes in appetite regulating neuropeptides, such as Npy (Connan et al., 2007; Lob et al., 2003; Bronsky et al., 2011; Støving et al., 2009; Gendall et al., 1999; Kaye et al., 1990) and in the serotonin (Bailer et al., 2005; Galusca et al., 2008) neurotransmitter system have been found a long time after weight restoration in restrictive anorexia nervosa. With respect to neurodegenerative disorders, reduced expression of the orexigenic Npy and its receptor has been associated with Alzheimer’s disease and other neurodegenerative disorders (Spencer et al., 2016; Davies et al., 1990; Minthon et al., 1990, 1996; Martel et al., 1990; Koide et al., 1995; Nilsson et al., 2001; Alom et al., 1990; Martignoni et al., 1992). Thus, in both ANR and neurodegenerative disorders the appetite regulatory system is detectably impacted, albeit in an as-yet poorly defined manner.

Just as in severe ANR cases, mice homozygous for the recessive mouse anorexia (*anx/anx*) mutation restrict their eating and become severely emaciated (Maltais et al., 1984). Thus, genetic analyses of the *anx* mutation might identify factors important for the maintenance of appetite regulatory circuitry and could provide insights towards the development of interventions targeting detrimental weight loss. In *anx/anx* mice, hypothalamic Npy-expressing (Npy+) neurons begin to degenerate at postnatal day (P)10-12 (Nilsson et al., 2011). By P19-22 most Npy+ processes and soma have disappeared, and *anx/anx* mice have become severely emaciated and perish (Maltais et al., 1984). In addition, 5-HT immunoreactive fibers innervate their target areas abnormally in *anx*/*anx* mice (Huynh et al., 2011; Son et al., 1994), and their body tremors, head weaving and hyperactivity can be improved by administration of 5-HT antagonists (Maltais et al., 1984). Currently, the primary molecular cause for neurodegeneration in *anx/anx* mice is unknown. Microarray-based analyses of P21 hypothalamic mRNA identified a 50% decrease in two genes: RNA polymerase II associated protein 1 (*Rpap1*) and NADH dehydrogenase (ubiquinone) 1 alpha subcomplex, assembly factor 1 (*Ndufaf1*), which reside in the *anx* critical interval (Lindfors et al., 2011). Ndufaf1 is a complex I assembly factor protein required for the first step of mitochondrial respiration. Lindfors et al. claimed that the 50% decrease in *Ndufaf1* impacted mitochondrial respiration sufficiently to be the primary *anx* causative event. However, further experimental support for this proposed mechanism, such as detection of an *anx*-specific molecular lesion affecting *Ndufaf1* or by genetic non-complementation or transgenic rescue experiments, is lacking. Hence, the role of *Ndufaf1* downregulation in *anx* phenotypes is debatable and the identity of the *anx* causative mutation or any other genetic factor that might contribute to its phenotypes is unknown.

Given the Npy+ neuron degeneration in *anx/anx* mice, we considered whether the TYRO3 protein tyrosine kinase 3 (*Tyro3*) gene, which has prosurvival roles in neuronal culture (Funakoshi et al., 2002; Zheng et al., 2009; Zhong et al., 2010) and resides within the *anx* critical interval, might contribute to *anx* phenotypes. Tyro3, along with its related Axl and Mer (also known as Mertk) receptors, is a member of the TAM family of receptor tyrosine kinases (reviewed in Hafizi and Dahlback, 2006; Linger et al., 2008; Lemke, 2013), and was previously also known as Rse (Mark et al., 1994), Sky (Ohashi et al., 1994), Brt (Fujimoto and Yamamoto, 1994), Tif (Dai et al., 1994), Dtk (Crosier et al., 1996), and Etk-2 (Biesecker et al., 1995). It is composed of a signal sequence, two immunoglobulin-like domains and two fibronectin type III repeats in its extracellular domains, a transmembrane domain and an intracellular tyrosine kinase domain. Upon activation by its ligands growth-arrest specific 6 (Gas6) or protein S, Tyro3 initiates homophilic dimerization, cross-phosphorylation, and downstream signaling through the PI-3K/AKT or Ras/ERK signaling pathways (reviewed in Linger et al., 2008; Lemke, 2013; Pierce and Keating, 2014). *Tyro3* is highly expressed in neurons (Funakoshi et al., 2002; Lai et al., 1994; Ohashi et al., 1994; Prieto et al., 2000). By contrast, *Axl* is expressed in the rostral migratory stream, whereas *Mer* mRNA expression is concentrated in glia (Ji et al., 2013). In mice (Duncan et al., 2003; Prasad et al., 2006; Maddox et al., 2011) and humans (Ostergaard et al., 2011; Shahzadi et al., 2010; Mackay et al., 2010; Brea-Fernandez et al., 2008), mutations in *Mer* cause retinal degeneration, but no other neurobiological phenotypes have been otherwise reported for mice lacking either *Axl* or *Mer* alone. However, in mice lacking both *Axl* and *Tyro3* the migration and survival of gonadotrophin release hormone (GnRH)-expressing neurons into the hypothalamus is compromised (Pierce et al., 2008). Moreover, loss of *Tyro3* accelerates retinal degeneration in *Mer^−/−^* mice (Vollrath et al., 2015). Triple *Tyro3*/*Axl*/*Mer* mutants exhibit cellular degeneration in the neocortex, hippocampus, cerebellum, and of rods and cones in the retina (reviewed in Ji et al., 2015). These studies illustrate the redundant reinforcing neuroprotective roles of TAM receptors, and open the door to the possibility that Tyro3 on its own might have significant neuroprotective potential.

Here, we identify a point mutation (C19T) in the signal sequence of Tyro3 (R7W-Tyro3), and detect a deficit in secondary platelet aggregation in *anx/anx* mice that resembles one previously reported in mice lacking *Tyro3*. We show that *Tyro3* is expressed in brain regions known to be affected by the *anx* mutation: the hypothalamic arcuate nucleus, the dentate gyrus and serotonergic neurons, and detect anomalies in RNA localization in *anx/anx* brains at P19. In the presence of wild-type *Tyro3* transgenes, but not of a C19T mutated *Tyro3* transgene (referred to here as *R7W-Tyro3*), the weight and lifespans of *anx/anx* mice was doubled relative to that of their non-transgenic *anx/anx* littermates and hypothalamic Npy+ neuron degeneration was delayed. Finally, we show that in the cerebella of transgenic mice, R7W-Tyro3 protein appeared mislocalized. Additional sequencing analyses revealed that the R7W-Tyro3 variant is present in AKR/J, FVB/NJ and NZW/lacZ mice, and thus R7W-Tyro3 is not the primary *anx* causative event, but rather is a strain-specific modifier of *anx* phenotypes. Taken together, these studies identify Tyro3 as having important neuroprotective roles in the appetite regulatory circuitry.

## RESULTS

### A point mutation in the signal sequence of *Tyro3* is associated with the *anx* phenotype

**Fig. 1.**
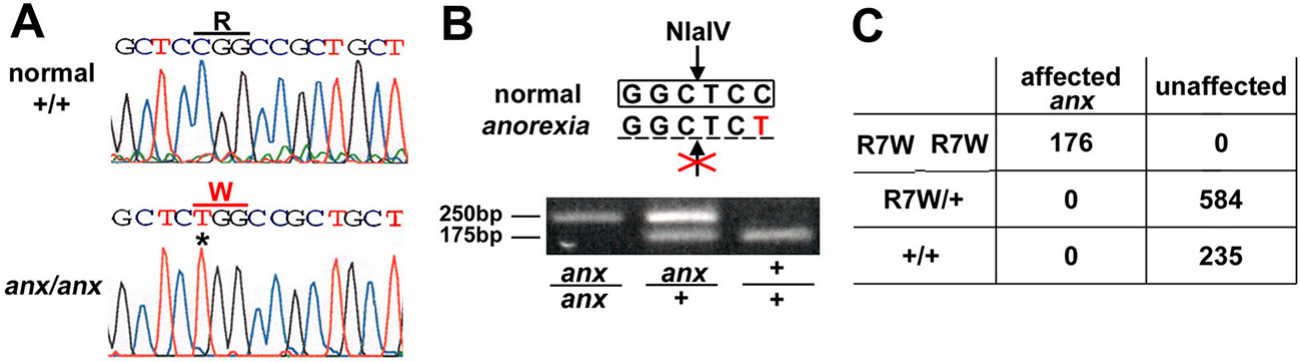
**Identification of a single nucleotide polymorphism in *Tyro3* associated with the *anx* phenotype.** (A) In mice carrying the *anx* mutation, a C-to-T mutation in *Tyro3* converts an arginine-to-tryptophan at amino acid 7 in Tyro3 protein (R7W-Tyro3) and (B) eliminates an *Nla*IV site. (C) Mice with the *anx* phenotype are homozygous for *R7W-Tyro3* (*n*=176), whereas *R7W-Tyro3/*+ (*n*=584) and *+/+* (*n*=235) mice appear normal.

The *Tyro3* gene is located within the *anx* critical interval, which we had refined to a 3.5 Mb region between D2Mit484 and D2Mit3 by using simple sequence length polymorphisms (SSLPs) to analyze 335 progeny (670 meioses) from heterozygous intercrosses on C57BL6/J (*n*=111) and *Mus molossinus/Ei* (*MOLF/EiJ*) (*n*=224) strain backgrounds (Fig. S1). Upon sequencing the exons and flanking intronic regions of *Tyro3*, we identified a cytosine-to-thymine (C19T) mutation in the signal sequence of *Tyro3* (Fig. 1A). This mutation converts an arginine to a tryptophan at the seventh amino acid position, and also eliminates an *Nla*IV restriction site (Fig. 1B), which we used in a PCR-based assay to genotype 995 additional animals. All 176 affected progeny were homozygous for the *R7W-Tyro3* mutation, whereas 819 unaffected animals were *R7W-Tyro3/+* or *+/+* (Fig. 1C). This sequence variant was absent in seven other inbred strains (C57BL6/J, C3H/HeJ, Balbc/J, MolfEi/J, CastEi/J, A/J, 129Sv/J) analyzed at the time. We identified two additional single nucleotide polymorphisms that led to non-synonymous changes (SNP rs47863852: Val811Leu and SNP rs13459232: Gly824Ser, ENSEMBL build GRCM38), but these were present in Tyro3 from some of the other strains sequenced here. Thus, we decided to test whether the R7W-Tyro3 variant might affect *anx* phenotypes.

### *anx/anx* mice exhibit platelet deficits similar to those reported in *Tyro3* nulls

**Fig. 2.**
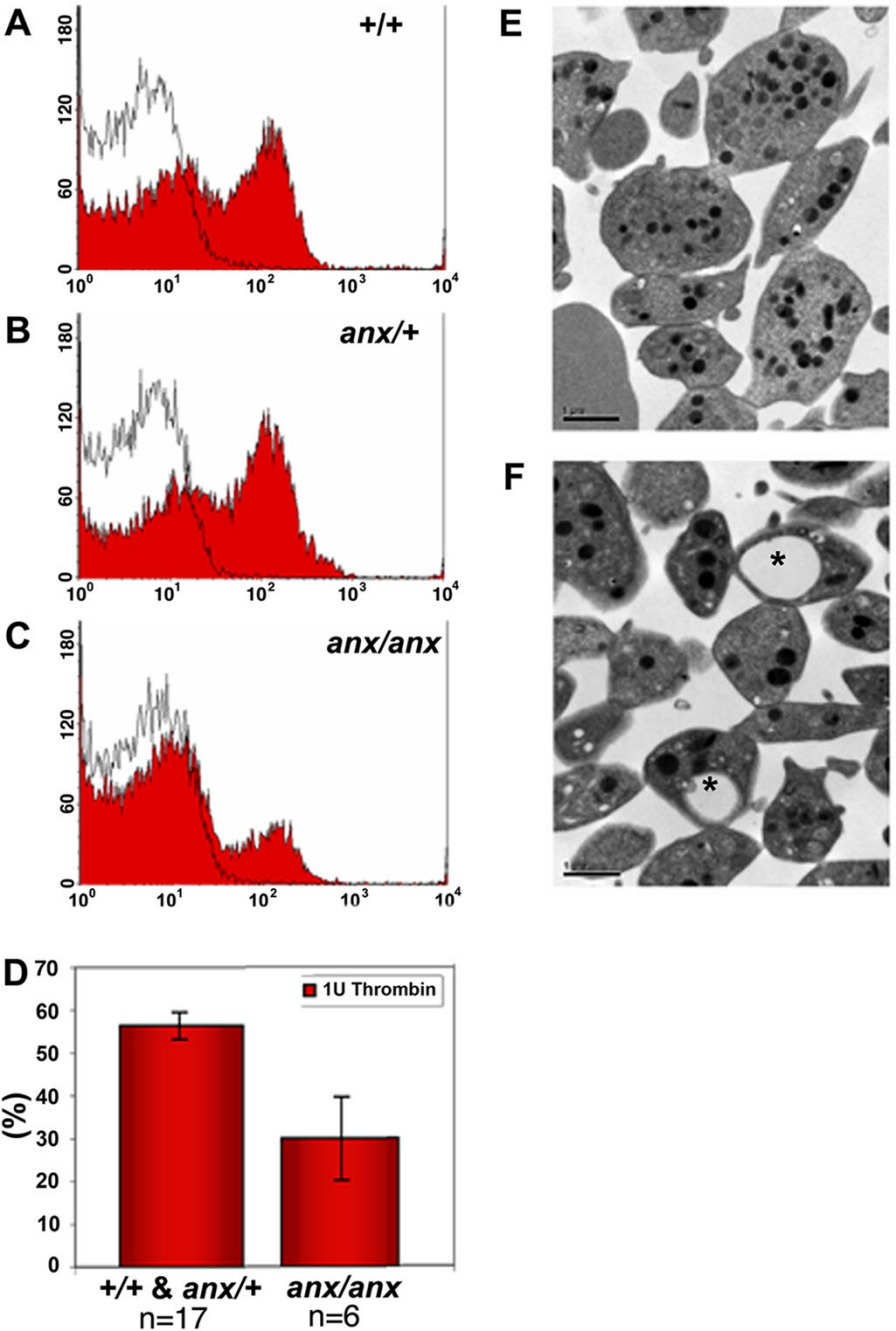
***anx*/*anx* mice show deficits in secondary platelet aggregation.** (A-C) The effect of (A) +/+, (B) *anx/+* and (C) *anx/anx* platelets on CD62 (P-selectin) expression, a marker for α-granule secretion, was detected using flow cytometry. After activation with thrombin, CD62 levels on the platelet surface were significantly reduced in (C) *anx/anx* platelets. Black lines represent resting platelets and red shading denotes thrombin-stimulated platelets. Representative examples of at least four independent experiments are shown. (D) The percentage of secondary platelet aggregation in +/+ and *anx*/+ animals was 56.4±3.2% (*n*=17) and *anx/anx* animals 23.9±9.8% (*n*=6) (Mann–Whitney Test, *P*=0.026). Data represented as mean±s.e.m. (E,F) Electron microscopy analyses revealed that (F) *anx/anx* platelets appeared normal, but often contain large vacuoles (indicated by asterisks) absent in (E) *+/+* platelets. The abnormal vacuoles were observed in 17.5±5.9% of *anx/anx* platelets (counted 57-63 platelets per replicate (*n*=4).

As a first step to test whether the R7W-Tyro3 variant might have functional biological consequences, we tested whether *anx/anx* mice exhibit deficits in platelet aggregation, as had been observed in *Tyro3^−/−^* mice (Angelillo-Scherrer et al., 2005; Gould et al., 2005). When we examined platelet aggregation in *anx/anx*, *anx/+* and *+/+* mice, we found that, upon activation with thrombin, P-selectin levels on the platelet surface were significantly reduced in *anx/anx* platelets (Fig. 2A-D). In platelets lacking Tyro3, Axl or Mer, reductions in P-selectin levels were observed upon ADP stimulation. The effect of thrombin on P-selectin levels has not been reported for *Tyro3^−/−^* platelets, nor did we examine the effect of ADP-stimulation on *anx*/*anx* platelets to further delineate the parallels of these aggregation deficits. Electron microscopy revealed that *anx/anx* platelet morphology appears normal, and dense and alpha granules are present in equivalent numbers per platelet surface area (Fig. 2E,F). However, *anx/anx* platelets appear smaller than +/+ and *anx/+* platelets (*P*<0.001) and 17.5±5.9% (mean±s.e.m.) (57-63 platelets per replicate; *n*=4) of *anx/anx* platelets contained large vacuoles, which were never observed in +/+ or *anx/+* platelets (Fig. 2F). Given the presence of these platelet deficits in *anx*/*anx* mice, we pursued the possibility that R7W-Tyro3 might further contribute to *anx* phenotypes.

### *Tyro3* expression in areas affected by the *anx* mutation and in *anx/anx* mutant brains

**Fig. 3.**
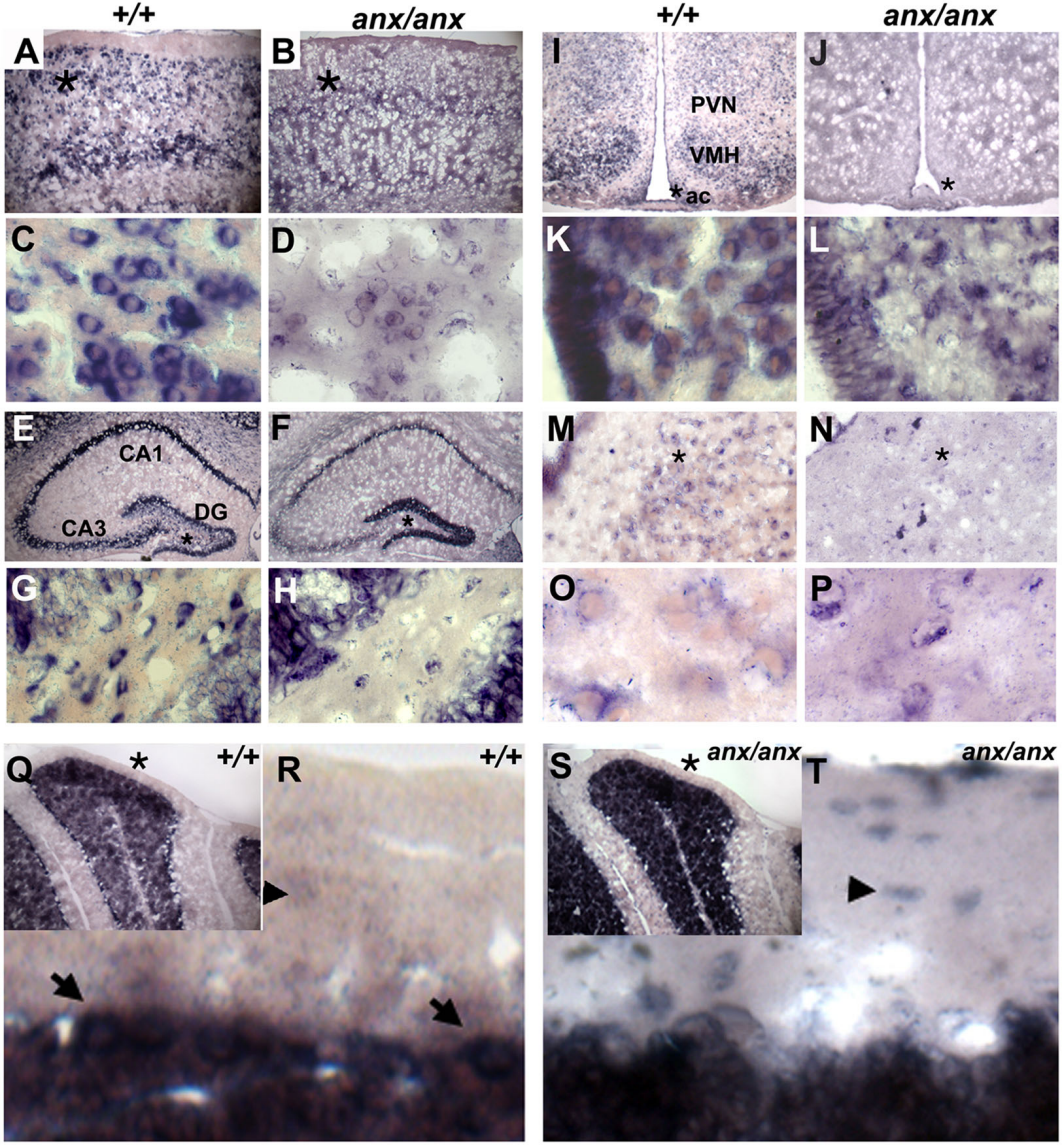
***Tyro3* RNA localization in +/+ and *anx/anx* brains at P21.** RNA *in situ* hybridization detected *Tyro3* RNA (A) in layers 2/3, 5 and 6 of *+/+*cortices. (B) Cortices of *anx/anx* mice show diffuse mutant (C19T) *Tyro3* RNA expression. (C) At high magnification, in the +/+ cortex, *Tyro3* is localized in soma at the base of processes. (D) In *anx/anx* mice, C19T-*Tyro3* RNA localization appears disorganized in the soma of cortical neurons. In the hippocampus of (E) +/+ and (F) *anx/anx* mice, *Tyro3* RNA is present in CA1, CA3, and the dentate gyrus (DG). (G) At higher magnification, *Tyro3* is expressed in cells within the granule cell and polymorphic layers of the +/+ DG. (H) In *anx/anx*, only a few cells in the granule cell and polymorphic layers of the DG express C19T*-Tyro3*. (I) In the +/+ hypothalamus, *Tyro3* is expressed in the median eminence, arcuate nucleus (ac), and ventromedial hypothalamus (VMH), and at lower levels in the paraventricular nucleus of hypothalamus (PVN). (J) In the *anx/anx* hypothalamus, C19T*-Tyro3* expression appeared so diffuse at low magnification that it is difficult to distinguish it in specific subregions. At higher magnification, (K) in the +/+ arcuate nucleus, *Tyro3* RNA is localized in processes emerging from neuronal soma, but (L) in the *anx/anx* arcuate nucleus, C19T*-Tyro3* RNA localization appears disorganized. *Tyro3* is expressed in neurons within the dorsal raphe nuclei of (M) +/+ and (N) *anx/anx* mice. At high magnification, *Tyro3* RNA is seen at the edges of (O) +/+ neuronal soma and in their emerging processes, but appears disorganized in (P) *anx/anx* neurons within the raphe nuclei. (Q) In the +/+ cerebellum, *Tyro3* is expressed in granule cells and Purkinje cells, shown at higher magnification in (R) (marked with arrows). In the cerebellar molecular layer, *Tyro3*-expressing processes can be seen at high magnification. *Tyro3* RNA faintly outlines the soma of cells – likely cerebellar stellate and basket cells (marked with arrowheads in R). (S) In *anx/anx* cerebella, C19T variant *Tyro3* RNA is present in granule and Purkinje cells. (T) At higher magnification, mutant *Tyro3*-positive cell bodies (marked with arrowheads), which are likely stellate or basket cells, can be seen in the molecular layer. Mutant *Tyro3*-expressing processes are not apparent. Asterisks in A,B,E,F,I,J,M, N,Q and S indicate regions shown at higher magnification directly below each respective panel in C,D,G,H,K,L,O,P,R and T.

Next, we examined *Tyro3* expression in wild-type and *anx/anx* brains. Overall levels of wild-type and mutant *Tyro3* RNA were not affected, as determined by qRT-PCR and northern blot analyses performed on +/+ and *anx*/*anx* brains at P21 (Fig. S2A). Previously, *Tyro3* mRNA had been detected in the subventricular zone and layers 2/3, 5 and 6 of the cerebral cortex, the CA1 region of the hippocampus, the median eminence of the hypothalamus, and granule cells of the cerebellum in the adult mouse brain (Lai et al., 1994; Schulz et al., 1995; Gely-Pernot et al., 2012). Deficits in these regions have not been reported in *anx/anx* mice. Using RNA *in situ* hybridization on P10 and P21 wild-type brains, we confirmed *Tyro3* expression in these regions (Fig. 3A,E,Q; Fig. S3), but also detected *Tyro3* mRNA in areas affected by the *anx* mutation. *Tyro3* was strongly expressed in the arcuate nucleus, ventromedial nucleus and, at lower levels, in the paraventricular nucleus of the hypothalamus and in ependymal cells lining the third ventricle (Fig. 3I). In the hippocampus, *Tyro3* was expressed in CA1, but also CA3 and, in the dentate gyrus, intensely in the molecular layer and in distinct cells within the granule cell and polymorphic layers (Fig. 3E). *Tyro3* mRNA was present in neurons – likely 5-HT ones – located within all raphe nuclei (Fig. 3M). In the cerebellum, *Tyro3* expression was seen in granule and Purkinje cells, faintly outlining basket and stellate cells, and in processes in the molecular layer (Fig. 3Q,R).

At P10, the expression pattern of *Tyro3* appeared equivalent throughout the brains of *anx/anx* and +/+ animals (data not shown). However, at P21, *anx*/*anx* brains showed marked differences in *Tyro3* expression. At low magnification, mutated (C19T) *Tyro3* expression appeared overall more diffuse in specific cell populations and throughout the brain, and mislocalized in some regions (Fig. 3B,F,J,N,S). Most noticeably, in the dentate gyrus, mutant *Tyro3* was highly expressed in the molecular layers of +/+ and *anx/anx* mice, but relative to *+/+* littermates (Fig. 3E,G), the granule and polymorphic layers from *anx/anx* mice contain very few cells expressing C19T-mutated *Tyro3* (Fig. 3F,H). At higher magnification, subcellular localization of the mutated mRNA appeared aberrant in P21 *anx/anx* brains. Generally, *Tyro3* mRNA appeared excluded from the nucleus, concentrated instead to regions within the soma from which processes were extending and, at times, could be detected in processes. In *anx/anx* neurons, C19T variant *Tyro3* RNA appeared concentrated in soma and was often seen in aggregates (Fig. 3D,H,L,P). For example, in wild-type cerebella, *Tyro3* mRNA was seen in processes in the molecular layer and only faintly outlining presumptive stellate and basket cell neurons (Fig. 3Q,R). However, in *anx/anx* cerebella the mutant *Tyro3* mRNA was detected only minimally in processes and was concentrated in the soma of presumptive basket and stellate cells (Fig. 3S,T). In summary, *Tyro3* was expressed in all neuronal populations – specifically the arcuate nucleus of the hypothalamus, the dentate gyrus of the hippocampus and presumptive serotonergic neurons in the raphe nuclei – for which anomalies have been reported in *anx* homozygotes, and the localization of C19T variant *Tyro3* RNA seemed to be affected throughout the brains of P21 *anx/anx* mice.

### Rescue of *anx* phenotypes by *Tyro3* transgenes

**Fig. 4.**
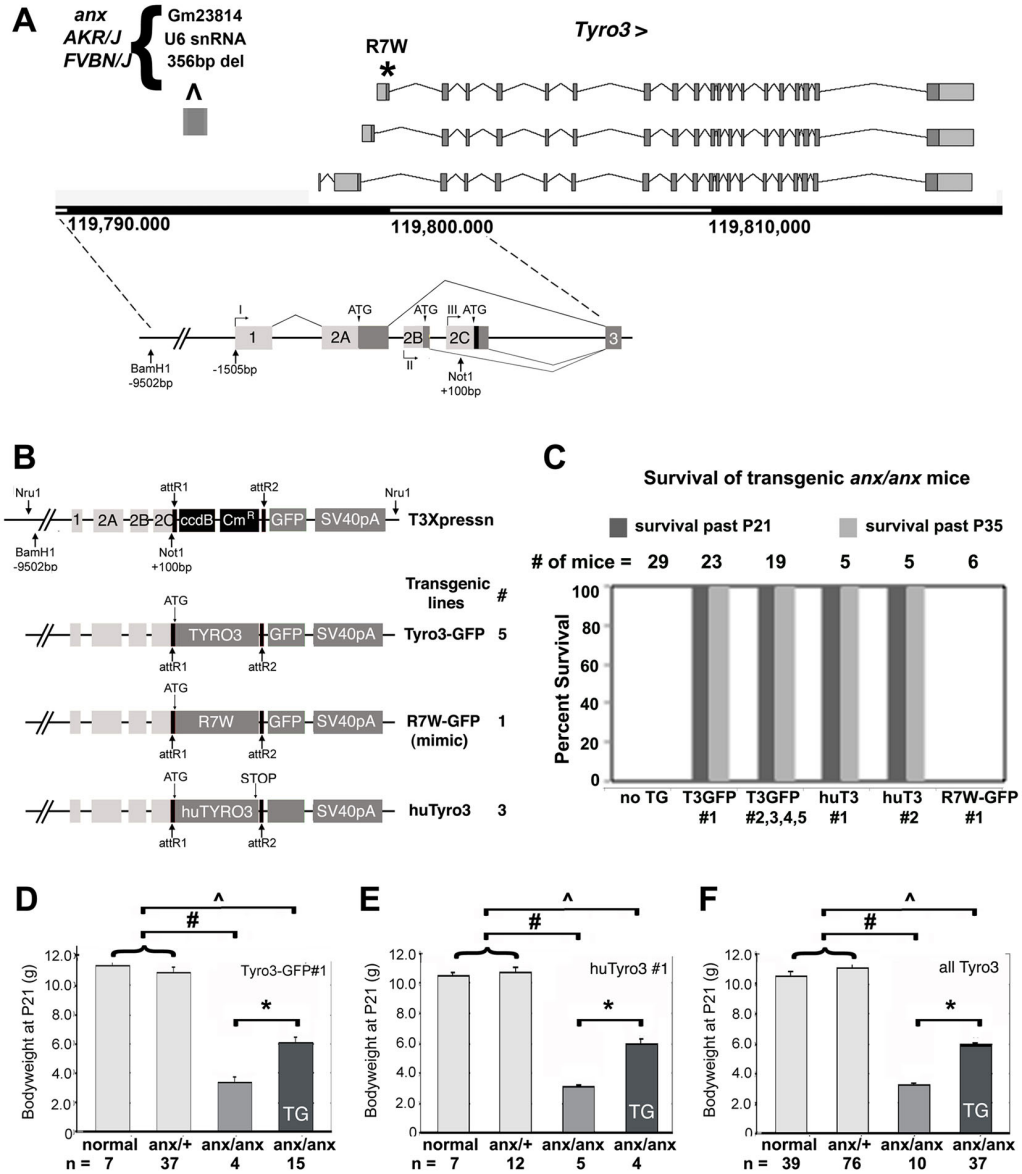
***anx/anx* lethality is delayed by *Tyro3* transgenes, but not by an *R7W-Tyro3* transgene.** (A) *Tyro3* transgenes were constructed using a 9.5 kb *Bam*HI-*Not*I fragment upstream of the translational start site located in exon 2c (adapted from Biesecker et al., 1995). The signal sequence of *TYRO3* is indicated in black. (B) The T3Xpressn vector was used to generate transgenic mice expressing mouse Tyro3-GFP, R7W-Tyro3-GFP and human TYRO3 (HuTyro3). The number of independent founder lines generated and analyzed is shown for each transgenic construct. (C) Analyses of survival of transgenic and non-transgenic *anx/anx* progeny. All *anx/anx* homozygous mice die by P21. In all mouse lines transgenic for Tyro3-GFP or huTyro3, transgenic *anx/anx* progeny survived past P35. *anx/anx* mice transgenic for R7W-Tyro3-GFP did not survive past P21. (D-F) Effect of *Tyro3* transgenes on bodyweight. For (D) Tyro3-GFP line #1, (E) HuTyro3 line #1, and (F) the sum total of all HuTyro3 and Tyro3-GFP transgenic lines, transgenic *anx/anx* mice weigh more than non-transgenic littermates at P21. Groups with significant differences relative to each other are indicated with brackets and a single asterisk for *anx/anx* versus *anx/anx; Tyro3* transgenic; # for *+/+* and *anx/+* vs *anx/anx*, and ^ for *+/+* and *anx/+* vs *anx/anx*; *Tyro3 transgenic*. *P*<0.01 for all groups by unpaired *t*-one-way ANOVA data represented as mean±s.e.m.

Next, we performed transgenic rescue experiments. Because phylogenetic sequence comparisons between *Tyro3* non-coding regions showed highest conservation within the first 9.5 kb upstream of exon 2c, we postulated that these regions might suffice to direct transgene expression in endogenous *Tyro3*-expressing domains, and used these regions from C57BL/6J genomic DNA to generate the T3Xpressn vector (Fig. 4A). We generated three varieties of transgenic mice: five lines expressing mouse Tyro3 protein C-terminally tagged with GFP (Tyro3-GFP), a line expressing mouse R7W-Tyro3 C-terminally tagged with GFP (R7W-Tyro3-GFP) and three lines expressing untagged human TYRO3 (HuTyro3) (Fig. 4B). The R7W-Tyro3 expression constructs were identical to the normal Tyro3 cDNA constructs except for the C19T polymorphism. GFP-tagged lines can facilitate tracking protein localization, but the GFP tag might compromise protein function. Untagged human TYRO3 should have full protein function, and its RNA can be distinguished from endogenous murine *Tyro3*. Tyro3-GFP and R7W-Tyro3-GFP protein localization in P21 +/+ brain regions recapitulated endogenous *Tyro3* RNA expression, as shown for the hypothalamus (Fig. S4) and serotonergic neurons (Fig. S5). In the arcuate nucleus and median eminence, a subset of Tyro3-GFP- and R7W-Tyro3-GFP-expressing neurons co-express Npy (Fig. S4). In the hindbrain raphe nuclei, Tyro3-GFP- and R7W-Tyro3-GFP-expressing neurons express the 5-HT marker tryptophan hydroxylase 2 (Tph2) (Zhang et al., 2004), thus identifying them as 5-HT neurons (Fig. S5). +/+ and *anx/+* mice transgenic for HuTyro3 or Tyro3-GFP were indistinguishable from non-transgenic *+/+* and *anx/+* littermates. Tyro3 transgenic wild-type mice showed no adverse effects with regards to viability, fertility and overall health.

Notably, all *anx/anx* mice transgenic for Tyro3-GFP or HuTyro3 showed mild or no neurobiological *anx*-related behaviors such as ataxia, shaking and headweaving at P21, and survived at least until P35, as shown for all Tyro3-GFP and HuTyro3 transgenic lines (Fig. 4C; *P*<0.01). By contrast, the *R7W-Tyro3-GFP* transgene, even though expressed at levels similar to some of the rescuing *Tyro3* transgenes (Fig. S2B), could not delay onset of the behavioral phenotypes by P12-15 or extend the lifespans of *anx*/*anx* mice past P21 (Fig. 4C).

At P21, all Tyro3-GFP and HuTyro3 transgenic *anx/anx* animals had significant body weight increases compared with non-transgenic *anx/anx* littermates. For example, *anx/anx* mice transgenic for Tyro3-GFP#1 or HuTyro3 line#1 had an average increased body weight of 83.5±8.2% or 92.8±8.5%, respectively, relative to their non-transgenic *anx/anx* littermates (*P*<0.01, one-way ANOVA; Fig. 4D,E). On average Tyro3-GFP and HuTyro3 transgenic *anx/anx* mice showed an 82.3±3.6% increase in body weight at P21 (Fig. 4F).

**Fig. 5.**
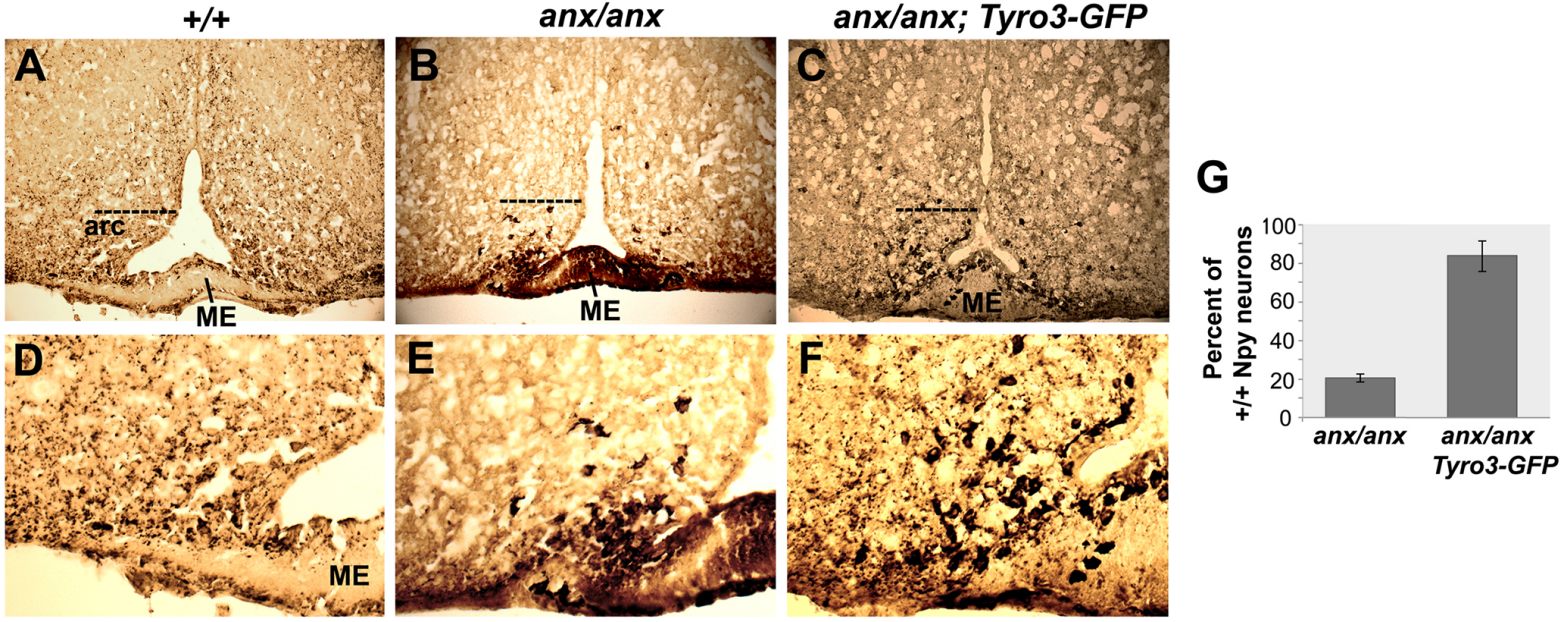
**Rescue of Npy+ neuron abnormalities of *anx/anx* mice at P19.** Npy+ neuronal abnormalities are ameliorated in *Tyro3-GFP*; *anx/anx* transgenic mice as detected by immunoperoxidase enhanced with silver staining with an anti-Npy antibody, which stains Npy+ neurons and processes black. (A,D) In P19 +/+ mice, Npy+ neurons are located in the arcuate nucleus (arc) and send processes to the paraventricular nucleus. A monolayer of Npy+ cells also lines the inside and outside of the median eminence (ME). (B,E) In P19 *anx/anx* mice, only a few Npy+ neurons are present in the arcuate nucleus, and Npy+ processes extending towards the PVN cannot be readily detected. In addition, some Npy+ cells are located abnormally in the median eminence (ME). (C,F) In Tyro3-GFP transgenic *anx/anx* mice, Npy+ neurons are mostly distributed throughout the arcuate nucleus and Npy+ puncta can be detected extending towards the PVN. D-F show higher magnification views of A-C, respectively. Dotted line in A-C demarcates the upper limit of the Npy+ soma included in the counts. (G) For *anx/anx* and *anx/anx*; *Tyro3-GFP* mice the percentage of Npy+ soma within the arcuate nucleus at the level of the median eminence relative to wild-type littermates are shown. Mice carrying *Tyro3-GFP* transgene (line #1) were analyzed. Cell counts were obtained from three sections with highest number of Npy+ neurons per animal and *n*=3 animals per genotype. Pairwise comparisons were performed using the Wilcoxon signed rank test; *P*<0.05.

Because Npy+ neuron degeneration has been linked to appetite deregulation in *anx/anx* mice (Nilsson et al., 2011), we examined the effects of the *Tyro3-GFP* transgene on Npy+ neurons in *anx*/*anx* mice. Normally, Npy+ neurons are found in the arcuate nucleus and innervate the parvocellular and magnocellular paraventricular nucleus of hypothalamus (PVN) and dorsomedial nucleus of the hypothalamus, as detected by silver-enhanced immunoperoxidase staining (Fig. 5A,D). At P19, the percentage of Npy+ soma present in the arcuate nucleus of *anx/anx* mice had been reduced to 20.2± 1.8% relative to wild-type littermates, whereas in *Tyro3-GFP#1*; *anx/anx* mice 83.58±7.9% were still present. Moreover, at P19, Npy-immunoreactive cell bodies are consistently clustered aberrantly within and adjacent to the median eminence in *anx/anx* mice. In all *anx/anx* mice examined no punctate Npy staining suggestive of Npy localization in processes could be seen extending out of the arcuate nucleus towards the PVN or dorsomedial nucleus of the hypothalamus (Fig. 5B,E). In *Tyro3-GFP#1*; *anx/anx* mice, Npy+ soma were distributed throughout the arcuate nucleus and punctate Npy staining suggestive of Npy+ processes extended towards and into the PVN as in +/+ mice (Fig. 5C,F). However, qualitatively, Npy immunoreactivity still appeared stronger in *Tyro3-GFP*; *anx*/*anx* neuronal soma than in those of +/+ mice. Thus, in the presence of the *Tyro3-GFP* transgene, more Npy+ neurons and Npy+ processes can be detected within the arcuate nucleus in P19 *anx*/*anx* mice an observation consistent with a delay in neurodegeneration. This increase in Npy-containing processes and hence Npy signaling is likely responsible for the increased weight gain seen in *anx/anx*; *Tyro3-GFP* mice. In summary, rescue of these *anx* phenotypes by *HuTyro3* and *Tyro3-GFP* transgenes, but not the *R7W-Tyro3* transgene, suggest that the C19T *Tyro3* mutation might contribute to the *anx* phenotypes.

### Effects of the R7W-Tyro3 variant

To understand the possible consequences of the C19T mutation on Tyro3 localization and function, we examined whether the mutation impinged on signal sequence function *in vitro*. Point mutations within signal sequences can impact protein secretion and function in a variety of ways. The Signal P program (www.cbs.dtu.dk/services/SignalP) (Bendtsen et al., 2004), which examines amino acid sequences for characteristics of protein signal sequences (Bendtsen et al., 2004), did not predict that *R7W-Tyro3* would substantially alter the signal sequence cleavage site (Fig. S6). Signal sequence mutations can also affect translocation from the cytoplasm to the endoplasmic reticulum and post-translational modifications, including N-glycosylation, that occur during protein secretion. In accordance with predictive analysis, when we assessed proteolytic processing of *in vitro* transcribed and translated Tyro3 and R7W-Tyro3 proteins mixed with signal peptidase from canine pancreatic rough microsomes, the extent of signal sequence cleavage and glycosylation of the R7W-Tyro3 protein seemed equivalent to that of Tyro3 (Fig. S7). Thus, the C19T mutation in *Tyro3* does not impact signal sequence function *in vitro*.

**Fig. 6.**
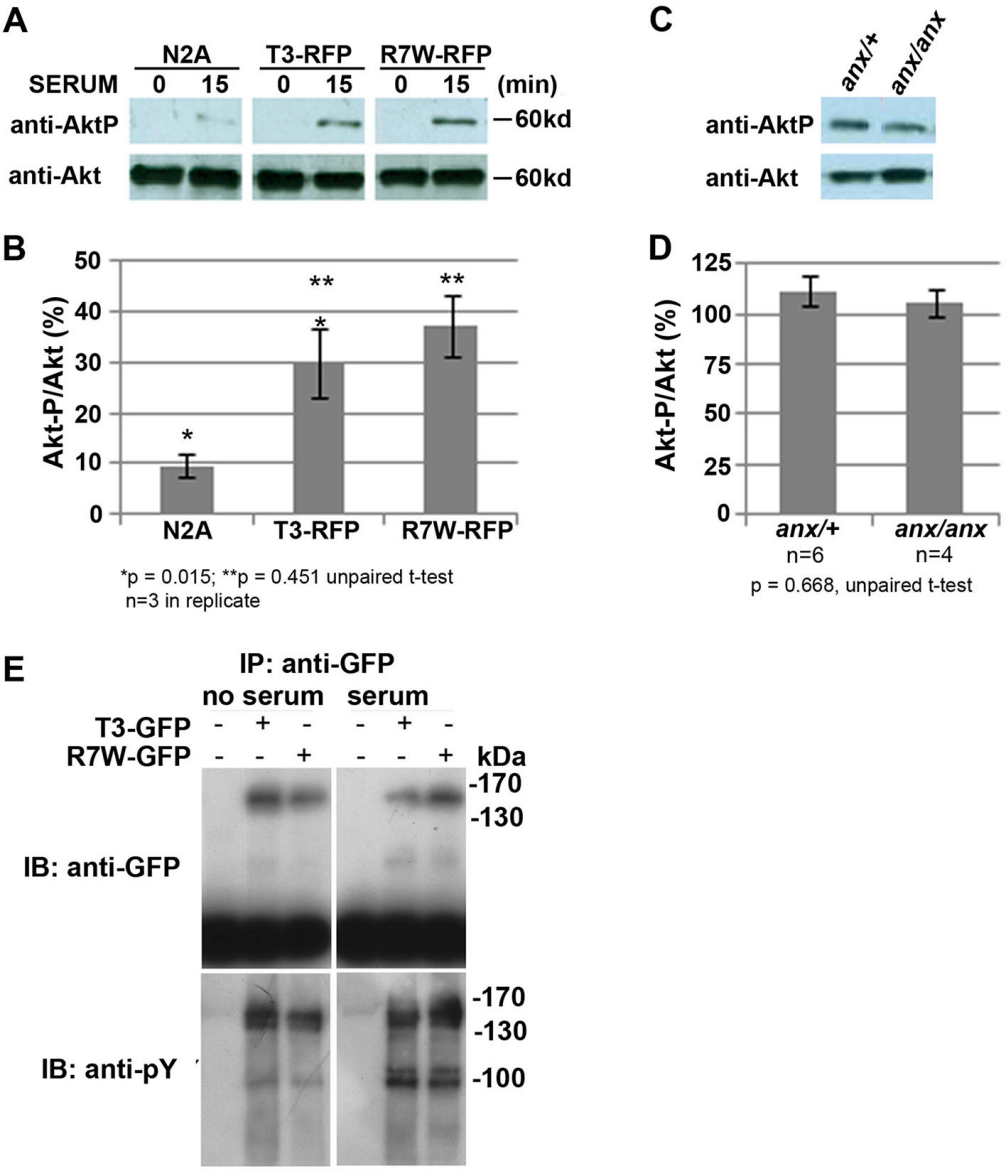
**R7W-Tyro3 and Tyro3 affect Akt phosphorylation indistinguishably in cell culture and in *anx/anx* cerebella.** Stable transfection of R7W-Tyro3 and Tyro3 results in equivalent Akt phosphorylation in response to serum. (A) Phosphorylation of Akt in response to 15 min exposure to serum after 12 h serum starvation in N2A cells stably expressing equivalent amounts of either Tyro3-RFP or R7W-Tyro3-RFP. (B) Percentage of phosphorylated Akt in response to serum. (C,D) Akt is phosphorylated to a similar extent in *anx/anx* and *anx/+* cerebella. (C) Western blot detects equivalent levels of Akt-P in cerebella of *anx/+* and *anx/anx* P10 mice. (D) The percentage of phosphorylated to unphosphorylated Akt is shown. (E) Serum-activated tyrosine phosphorylation of Tyro3-GFP and R7W-Tyro3-GFP seems equivalent in transiently transfected COS7 cells. In the absence and presence of serum, immunoprecipitation of Tyro3-GFP and R7W-Tyro3-GFP was carried out with an anti-GFP antibody. Subsequent analysis with an anti-phosphotyrosine antibody revealed no differences in tyrosine phosphorylation as indicated by equivocal banding patterns. *P<0.05, **P<0.01 by unparied t-test; data represented as mean±s.e.m.

In the presence of GAS6, an established Tyro3 ligand present in serum, Tyro3 activation leads to Akt phosphorylation (Lan et al., 2000; Zhong et al., 2010). Therefore, we examined whether serum-dependent Akt phosphorylation was affected *in vitro* and *in vivo*. We detected no differences in Tyro3- or R7W-Tyro3-dependent Akt phosphorylation in the absence or presence of serum in transiently transfected COS7 cells, in stably transfected Neuro2A cells or in protein extracts from +/+ and *anx/anx* cerebella or hippocampi (Fig. 6 and data not shown). In the absence or presence of serum, immunoprecipitation of Tyro3-RFP and R7W-Tyro3-RFP using an anti-RFP antibody and subsequent immunoblot analysis with an anti-phosphotyrosine antibody also revealed no detectable differences in tyrosine phosphorylation (Fig. 6). Thus, signaling function does not appear to be overtly compromised by the C19T *Tyro3* mutation.

**Fig. 7.**
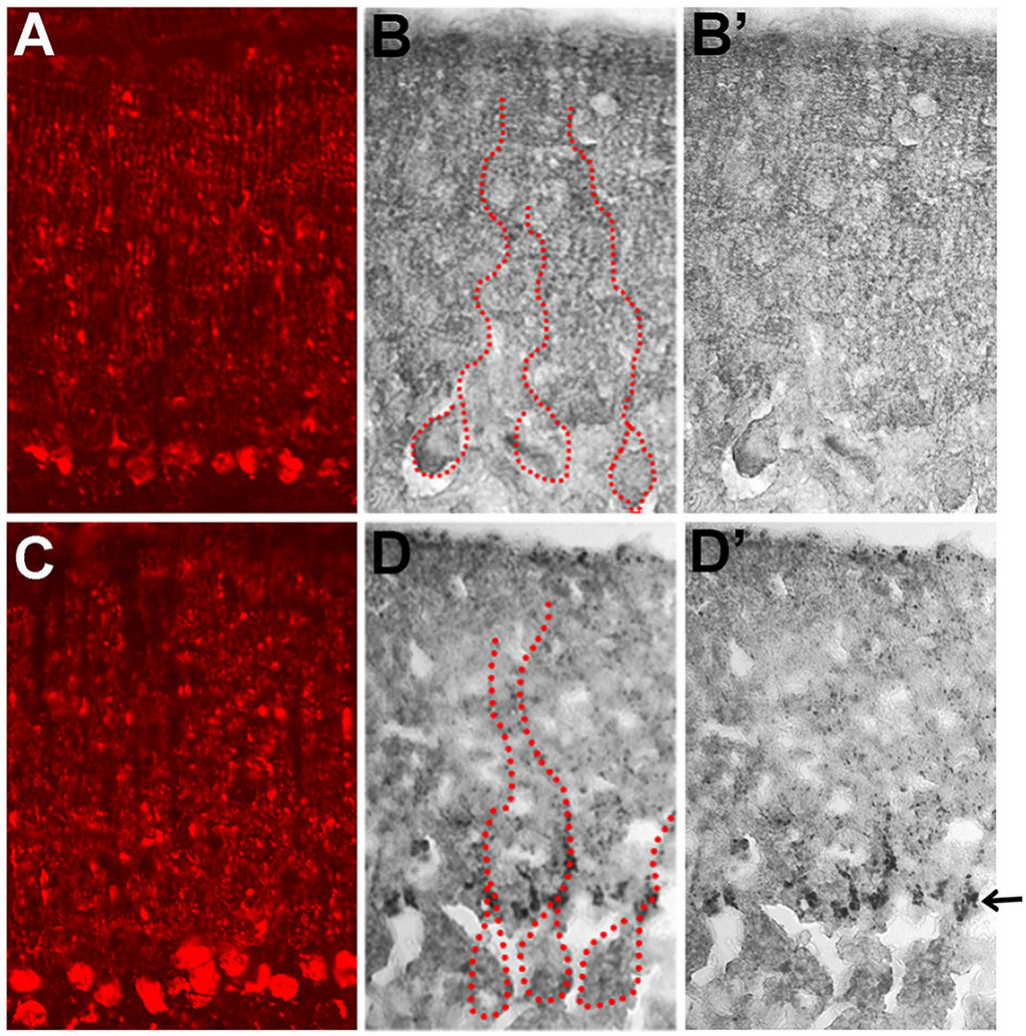
**Tyro3-GFP and R7W-Tyro3-GFP protein localization in the molecular layer of the cerebellum of transgenic +/+ mice.** Cerebellar Purkinje cells appear indistinguishable in (A) Tyro3-GFP and (C) R7W-Tyro3-GFP transgenic, +/+ mice as detected by immunofluorescence with anti-calbindin antibody. (B,B′) Silver-enhanced immunohistochemical analyses using an anti-GFP antibody detected Tyro3-GFP protein distributed in fine puncta throughout the molecular layer of the cerebellum. (D,D′) R7W-Tyro3-GFP protein is aggregated at the bottom of the dendritic shaft (marked by an arrow) emerging from Purkinje cell bodies. Larger puncta are seen along presumptive primary dendritic shafts. Some large puncta are also gathered near the top of the molecular layer. In B and D, the cell bodies and main shafts of several Purkinje cells are outlined with dotted red lines.

When we examined whether Tyro3-GFP and R7W-Tyro3-GFP protein localization differed in transiently transfected Neuro2A, COS7 and HEK293T cells or in stably transfected Neuro2A cells, we found that in all instances their subcellular localization appeared indistinguishable (Fig. S8 and data not shown). Using immunofluorescence with an anti-Tyro3 antibody on cultured cortical and hippocampal neurons from *anx/anx* and *+/+* littermates, endogenous R7W-Tyro3 and Tyro3 localization also appeared indistinguishable (Fig. S9). However, when we used silver-enhanced immunohistochemistry with an anti-GFP antibody to survey Tyro3-GFP and R7W-Tyro3-GFP protein distribution in the brains of P10 transgenic +/+ mice, we detected distinctly different localization patterns in cerebella (Fig. 7). At P10, *Tyro3-GFP; +/+* and *R7W-Tyro3-GFP; +/+* Purkinje cells appear normal, as determined by calbindin immunofluorescence. Tyro3-GFP protein was distributed in tiny puncta throughout the molecular layer of transgenic +/+ cerebella (Fig. 7B,B′). However, R7W-Tyro3-GFP protein was predominantly found in Purkinje cell soma and in the major shafts extending from the soma in large, sparse puncta (Fig. 7D,D′). The stereotypic organization and large dendritic arbors of Purkinje cells facilitated detection of this mislocalization, which was otherwise not obvious in other brain regions. Thus, the R7W-Tyro3 variant causes protein mislocalization in the postnatal cerebellum.

### R7W-Tyro3 is a strain-specific modifier of the *anx* phenotype

The anorexia mutation arose spontaneously on a mixed Swiss Webster/*Mus poschiavinus* background (Maltais et al., 1984) and has subsequently been maintained on a C57Fe/a background. Recently whole genome sequence has become available for 17 inbred mouse strains in addition to C57BL/6J (Yalcin et al., 2012; Keane et al., 2011; Wong et al., 2012). We found that the R7W-Tyro3 variant is also present in FVB/NJ, AKR/J and NZW/lacJ strains, which are related to the Swiss Webster strain. As none of these strains exhibit *anx*-like phenotypes, the R7W-Tyro3 variant cannot be the primary genetic lesion responsible for *anx* phenotypes. All three of these strains also share other sequence variations, such as the deletion of a U6 pseudogene in the *Tyro3* promoter region (Fig. 4). This pseudogene is present in all *Tyro3* and *R7W-Tyro3* transgenic constructs, and hence does not contribute to the rescue of *anx* phenotypes observed in our *Tyro3* transgenic lines. All of these observations indicate that the *anx* mutation arose on an ancestral Swiss Webster-related background and that Tyro3 acts as a strain-specific linked modifier of *anx* phenotypes.

## DISCUSSION

Our study reveals *Tyro3* as the first validated, linked gene that contributes to *anx* phenotypes and can delay hypothalamic neurodegeneration in *anx/anx* mice. As aforementioned, previous microarray analysis of the degenerating arcuate nucleus from P21 *anx*/*anx* mice (Lindfors et al., 2011) revealed twofold differences in the levels of *Ndufaf1* and *Rpap1*. We found that cDNAs of both *Ndufaf1* and *Rpap1* are 100% identical to those of AKR/J mice. We did find a shared intronic SINE insertion within the *Ndufaf1* gene and a 222 bp deletion located 1296 bp upstream of the *Rpap1* transcriptional start site in both *anx/anx* and AKR/J mice and these differences could conceivably interfere with *Ndufaf1* and *Rpap1* transcription, respectively. Furthermore, whereas mitochondrial dysfunction can lead to neurodegeneration, abnormal mitochondrial respiration and localization can also be a consequence of neurodegeneration (Martin, 2012). Thus, although it remains a possibility that decreases in *Rpap1* and/or *Ndufaf1* levels might influence *anx* phenotypes, in contrast to *Tyro3*, at present any direct experimental support for the involvement of either is lacking.

Our findings reveal roles for Tyro3 in maintaining Npy+ neurons within the appetite regulatory circuitry. Unlike *anx*/*anx* mice, no abnormalities in size or appetite have been reported for *Tyro3*^−/−^ or TAM^−/−^ mice. A series of studies in which AgRP neurons were ablated at different times and over varying timeframes might help explain why *anx*/*anx* mice show acute hypothalamic deficits and why, if such deficits are present in *Tyro3*^−/−^ or TAM^−/−^ mice or other mouse mutants, they might have been missed. AgRP/Npy+ neuron ablation that occurs acutely at birth or gradually over a period of a few months leads to only mild decreases in weight (Bewick et al., 2005; Xu et al., 2005). By contrast, acute AgRP/Npy+ neuron ablation in adult mice (Gropp et al., 2005; Luquet et al., 2005) or in *anx/anx* mice starting at P10-P12, a time by which arcuate nucleus neuron projections have formed functional connections with other hypothalamic regions (Bouret et al., 2004), inhibits feeding behavior and causes starvation. Thus, appetite regulatory circuitry can adjust and compensate for gradual adult or acute neonatal loss, and gradual Npy+ neuron loss in Tyro3^−/−^ or TAM^−/−^ mice would have gone unnoticed. Considering that other genes within the *anx* critical interval do not play known roles in Tyro3 signaling, it seems likely that Tyro3 boosts neuronal survival in general rather than by interacting specifically with the as-yet-unknown primary *anx* causative gene.

### Tyro3 and restrictive anorexia nervosa

To date no unequivocal, well-replicated genes or loci associated with ANR have been identified in humans (reviewed in Yilmaz et al., 2015). With respect to neurotrophic factors and tyrosine kinase receptor signaling, candidate gene approaches have obtained mixed results for linkage of the rs6265 (V66M) polymorphism in brain-derived neurotrophic factor (*BDNF*) with ANR (Dardennes et al., 2007; Ribasés et al., 2005, 2004; Ando et al., 2012; Brandys et al., 2013; Rybakowski et al., 2007). In the case of appetite-regulating neuropeptides, so far missense mutations in POMC have been reported in one individual with ANR, but no associations with common variants have been detected in ANR populations (Yilmaz et al., 2014). Furthermore, several genome-wide linkage studies found association of the serotonin receptor 1D and opiod receptor delta 1 with ANR (Bergen et al., 2003; Brown et al., 2007). Considering that body mass index remains low in recovered individuals with ANR, and ANR predominantly affects females, a genome-wide association meta-analysis cross-referencing these traits uncovered possible associations with, amongst other genes, *CTBP2* and *NBEAL1* (Hinney et al., 2016). Both *CTBP2* and *NBEAL1* show altered hypothalamic expression in response to fasting and diet-induced obesity and are postulated to act upon or at synapses. Finally, epoxide hydrolase 2 (*EPHX2*) emerged as a candidate for ANR associated with increased depression and anxiety in a high-throughput sequencing study (Scott-Van Zeeland et al., 2014). The paucity of known genetic factors likely reflects the heterogeneity among ANR cases and the genetic complexity of ANR specifically and psychiatric disorders in general. Because onset of ANR is accompanied by a range of psychiatric manifestations, including anxiety, body dysmorphic disorder, and often heightened sensitivity to negative stimuli, which are not of hypothalamic origin, and because these often persist to some degree even upon recovery, it seems unlikely that hypothalamic degeneration serves as an initiating event in ANR. Nonetheless, should any hypothalamic neurodegeneration occur in ANR – possibly as a secondary effect – normal Tyro3 signaling might offset this and perhaps improve chances of recovery.

### Tyro3 and appetite dysregulation in neurodegenerative disorders

Whether hypothalamic degeneration accompanies the ingestive dysfunctions observed in some patients with neurodegenerative disorders, such as amyotrophic lateral sclerosis (ALS), which, in familial cases, is often caused by mutations in Cu/Zn superoxide dismutase (*SOD1*) or hexanucleotide repeat expansions in chromosome 9 open reading frame 72 (*C9ORF72*), is unknown. The observation that ALS shows great variability in disease onset and progression even within families (Penco et al., 2011; Fogh et al., 2007; Kim et al., 2007) has fueled the pursuit of genetic disease modifiers in humans, mice, flies and yeast (Couthouis et al., 2011; Zhang et al., 2015; reviewed in Casci and Pandey, 2015; Paul and Gitler, 2014). As TAM receptors are unique to chordates, Tyro3 would not have been identified in invertebrate or yeast-based screens. A few receptor tyrosine kinase (RTK) signaling pathways, which promote cell survival and act protectively on motor neurons both in *in vivo* and *in vitro* ALS models, have been uncovered and include glial-derived neurotrophic factor (GDNF), ciliary neurotrophic factor (CNTF), and insulin-like growth factor 1 (IGF-1) (Tovar et al., 2014; Kasai et al., 2011; Riboldi et al., 2011). Moreover, for variants in the VEGFA and EphA4 RTK signaling pathways, findings from animal models align well with their linkage to disease severity in individuals with ALS (Lambrechts et al., 2003; Van Hoecke et al., 2012). Given our findings, it might be warranted to examine whether Tyro3 variants are linked with the occurrence and severity of ingestive dysfunction in neurodegenerative disorders or, for that matter, with the general onset and severity of ALS or other degenerative disorders in general.

### Tyro3 as a strain-specific modifier

In some instances, some of the other mouse strains that carry the R7W-Tyro3 variant have shown enhanced vulnerability to neurodegenerative disorders. For example, mice, which lack the SMN1 RNA binding protein and act as a model for human spinal muscular atrophy (SMA), survived twice as long on a pure C57BL/6N background than on the R7W-Tyro3-carrying FVB/N background (Ackermann et al., 2013). Moreover, mice lacking the amyotrophic lateral sclerosis 2 (*Als2*) gene exhibited a shorter life span than wild-type littermates when they were on a FVB/N than on a C57BL6/J background (Hadano et al., 2010). Thus, the R7W-Tyro3 variant might be one of the factors that contribute to increased disease susceptibility in these instances.

### Possible consequences of the R7W-Tyro3 variant

Although bioinformatic analysis did not predict an appreciable impact on Tyro3 processing and we did not observe dramatic changes in the glycosylation state of the extracellular domain or localization of the mutant protein in cell culture, the R7W-Tyro3 variant appears to have consequences *in vivo*. Often, the biochemical effects of signal sequence mutations that are associated with human disorders correlate well with the disease state, as shown for prepro-parathyroid hormone (PTH) (Arnold et al., 1990). However, sometimes, even though a human disease is apparent, signal sequence function seems normal in *in vitro* assays. For example, a subtle mutation in the signal sequence of the cell surface calcium-sensing receptor (CASR) resulted in less protein at the plasma membrane, but the *in vitro* co-translational processing of the CASR mutant protein was unaffected (Pidasheva et al., 2005). Nonetheless the individual carrying this mutation, along with others carrying more dramatic mutations in the signal sequence of CASR, suffered from hypocalciuric hypercalcemia. Another example is a mutation in the signal sequence of thyroxin-binding globulin (TBG), which is associated with a partial deficiency of TBG-Allentown. Even though the Signal P program predicted no change in cleavage site, TBG was lowered in the blood of individuals with this deficiency (Fingerhut et al., 2004). Thus, C19T mutation in *Tyro3* might compromise signal sequence function subtly in general or significantly in specialized, long-lived cell types, such as neurons.

Another possibility for the apparent lack of impact of the R7W-Tyro3 variant on *in vitro* signal sequence function is that it impacts RNA-sequence-based functions. Although, conventionally, the main roles of signal sequences have been based on their peptide sequences, the RNA sequence of the signal sequence coding region of some secretory proteins can activate nuclear mRNA export (Palazzo et al., 2007). Consistent with the possibility of an RNA-based mechanism, comparison of all sequenced *Tyro3* orthologues revealed that the cytosine mutated in the C19T *Tyro3* mutation is invariant and located within a larger conserved 21 nucleotide sequence. Current programs designed to identify RNA binding protein sites (RBPDB: http://rbpdb.ccbr.utoronto.ca/index.php and RegRNA: http://regrna.mbc.nctu.edu.tw/html/prediction.html) did not score disruption or creation of a binding site for a known RNA binding protein (RBP) by the C19T *Tyro3* mutation. Moreover, the RNA binding profiles for some neuronally important RNA binding proteins (RBPs), such as FMRP1 (Miyashiro et al., 2003; Darnell et al., 2001, 2005), Nova (Ule et al., 2003), TDP-43 (Sephton et al., 2011), RbFox1 (Lee et al., 2016) and SRRM4/nSR100 (Irimia et al., 2014), have begun to be determined, but none have recovered wild-type *Tyro3* mRNA. Endogenous Tyro3 protein is localized in neuronal processes (Fig. S9) (Prieto et al., 2007). Many regulatory elements known to govern localization of neuronal mRNAs, such as BDNF (An et al., 2008) and Arc (Rao et al., 2006), reside in 3′UTRs. However, the transgenic *Tyro3* expression constructs contain only *Tyro3* coding sequences and 154 nt of the 5′UTR, and the C19T *Tyro3* mutation resides near the translational start site. Taken together, these observations might be more in line with a disruption of translational regulation. Given that we could only observe protein and possible RNA mislocalization *in vivo* and not in culture, neuron-specific RNA-binding and/or translational regulatory factors – possibly ones dependent on neuronal activity – might be involved, and these might either be absent in cell culture or not have been activated under normal culture conditions. However, although an intriguing speculation, it is currently unknown whether an RNA-based mechanism – and perhaps a novel one – might play a role in R7W-Tyro3 mislocalization.

Regardless of mechanism, the mislocalization of R7W-Tyro3-GFP protein in the cerebella of wild-type mice does suggest that the R7W variant causes protein mislocalization *in vivo* and that, given that these mice appear normal, this effect is not due to overtly compromised neuronal health. The molecular mechanism by which the R7W-Tyro3 variant disrupts Tyro3 function(s) remains unknown, and given that its effect is only apparent *in vivo* in the mouse and most striking in *anx/anx* mice, identification and validation of such a mechanism is more challenging.

## CONCLUSION

In conclusion, here we have identified a mutation in the signal sequence of the Tyro3 tyrosine kinase receptor (R7W-Tyro3) as enhancing *anx* phenotypes, and shown that normal Tyro3 acts protectively to sustain the appetite regulatory circuitry in *anx/anx* mice. Whereas predictive programs and *in vitro* analyses could not predict or detect appreciable differences, the biological consequences of both the R7W-Tyro3 variant and normal Tyro3 are apparent *in vivo*. Furthermore, our findings suggest the existence of subtle, quite possibly often missed, mechanisms that might regulate protein and/or RNA subcellular localization. To date platelet function has not been systematically examined in ANR. Our observations suggest that, in the future, systematic analyses of platelet function in eating disorders and possibly other psychiatric and neurodegenerative disorders might prove useful and help categorize these into specific biological subtypes. Overall, our observations highlight the importance of analyses in the mouse or other model organisms as a testbed during the search for human disease modifiers and for genetic variants with measurable biological effects. Finally, enhancing Tyro3 expression or signaling could provide a strategy for counteracting deficits in the appetite regulatory circuitry that might contribute – in a primary or secondary manner – to the ingestive dysfunctions seen in ANR and some neurodegenerative disorders.

## MATERIALS AND METHODS

### Mice

*anx*/+ mice maintained on a B6C3Fe *a*/*a* hybrid background were obtained from Jackson Laboratories (Bar Harbor, Maine, USA) and crossed onto C57Bl6/J and Molf/EiJ backgrounds. To identify mice carrying the C19T-*Tyro3* mutation, polymerase chain reaction (PCR)-based assay using primers 5′-GATGGCGCTGAGGCGGAGCATG and 5′-CGCGGCCGGAGGTCTGGCAG, and subsequent digestion with *Nla*IV was used. All animal experiments were performed in strict adherence to guidelines for experimentation with laboratory animals set by the Canadian Animal Care Center (CACC).

### Platelet aggregation assays

Platelet aggregation assays were performed as in Angelillo-Scherrer et al. (2005). In brief, 1×10^6^ platelets per mouse were incubated with 1 U thrombin (Sigma) for 10 min at room temperature (RT), labeled with FITC-conjugated anti-P-selectin antibody (1:1000; BD Biosciences-Pharmingen Cat. no. 561849 and analyzed by flow cytometry using a FACs instrument and Cell Quest software. Platelet aggregation was analyzed for six or more mice of each genotype (*anx*/*anx*, *anx*/+, +/+) and data analyzed using the Mann–Whitney Test.

### Platelet electronmicroscopy

Isolated platelets were fixed with 4% paraformaldehyde and 2% gluteraldehyde in 0.1 M sodium cacodylate buffer pH 7.3. Sectioned samples were analyzed by Tecnai 20 transmission electron microscopy followed by data analysis using Excel Docu (Soft Imaging Systems). For morphologic analyses, 56-63 platelets from each of four *anx*/*anx* and three *anx*/+ and +/+ animals were analyzed. Data was analyzed using the Mann–Whitney Test.

### Quantitative RT-PCR

RNA from P19 cortices from *anx/anx*, *anx*/+ and +/+ mice was isolated using Trizol reagent (Invitrogen) according to manufacturer's instructions. cDNA was synthesized from total RNA using oligo-dT primers and the Superscript III First Strand Synthesis System (Invitrogen) according to manufacturer's instructions (2 µg of RNA used in 20 µl reverse-transcription reaction). Primers specific for *Tyro3* (5′-AATGCCGAGATTTACAACTACCTCAT-3′ and 5′-GTTCCATTCGACAGACACGTGAAGCTT-3′) and GAPDH (5′-TGTGAACGGATTTGGCCGTAT-3′and 5′-CATGTAGACCATGTAGTTGAG-3′) were used. Reactions were run with SYBR green PCR master mix (Invitrogen) in an AB 7500 fast real-time PCR system and relative gene expression levels were calculated using Sequence Detection System 2.2 Software (Applied Biosystems). Expression values were normalized relative to sample *Gapdh* mRNA expression. RNA from five *anx*/+ and +/+ and three *anx*/*anx* animals was analyzed. Data were analyzed using nonparametric *t*-tests and Mann–Whitney test.

### RNA *in situ* hybridization on sections

RNA *in situ* hybridization on sections was performed on brains perfused with 4% paraformaldehyde in diethyl-pyrocarbonate-treated 1×PBS and embedded in optimal cutting temperature medium (Sakura Finetek, Torrance, California, USA). Coronal sections of 14 µm were cut using the Leica VT1000 cryostat. RNA *in situ* hybridization was performed on tissue sections as described in Storm and Kingsley (1996). Sense and antisense probes were generated from a 588 bp *Hin*dIII fragment, containing the partial coding sequence of the Tyro3 intracellular kinase domain, cloned into pBluescript KSII. The sense probe was generated with *Xho*I digestion and T3 RNA polymerase transcription, the antisense probe upon *Eco*RI digestion and *in vitro* transcription with T7 RNA polymerase (Roche, Basel, Switzerland).

### Generation of transgenic animals

*Tyro3* and *R7W-Tyro3* cDNA was generated from RNA from +/+ and *anx*/*anx* brains using Superscript First-Strand Synthesis RT System (Invitrogen), amplified by real-time PCR, and inserted into pDONR201 with BP Clonase (Invitrogen). To generate an untagged human TYRO3 construct, human TYRO3 cDNA (generously provided by Dr Kensaku Mizuno, Tohoku University, Sendai, Japan) was inserted into filled-in *Sal*I sites of pDONR201. Using LR Clonase, these versions of *Tyro3* were cloned into the T3Xpressn vector. The T3Xpressn vector consists of 9.5 kb *Bam*HI-*Not*I fragment 5′ of and containing the transcriptional start site for signal sequence-containing exon 2c (Biesecker et al., 1995) and 154 nt of the 5′UTR, *attR* recombination sites, an in-frame C-terminal GFP, and Sv40 IVS polyA. Resulting expression constructs were digested with *Nru*I, purified with GeneClean (Qbiogene, Irvine, California, USA), and used to generate transgenic mice by standard pronuclear microinjection into FVBN/J oocytes. Positive transgenic mice were identified by Southern blot analysis and by a PCR-based assay using the following primers: GWTyro3-F1: GGGGACAAGTTTGTACAAAAAAGCAGGCTGCCGCCGATGGCGCTGAGGCGGAGCATGGGGTG and GWTyro3-R1: GGGGACCACTTTGTACAAGAAAGCTGGGTTAACTGCTACTGTGAGGCAGTAGCCCTTG.

### Immunostaining

In all cases, a minimum of three mice of a given genotype and stage were analyzed. Adult brain cryosections were blocked in 10% normal donkey serum (NDS) in PBS with 0.2% Triton X-100 (PBST) for 1 h at RT. Antibodies were diluted in 2% NDS PBST and incubated overnight at 4°C. Samples were washed and incubated with Cy3 or Alexa Fluor 488 secondary antibodies (1:500; Invitrogen) for 1 h at RT. Samples were mounted with Vectashield plus DAPI (Vector Labs).

For immunofluorescence, primary neuronal culture samples were fixed with 4% paraformaldehyde, permeabilized with 0.3% Triton X-100 PBS for 5 min at RT. All samples were blocked in histoblock [3% BSA, 1% BM blocking solution (0.01 M maleic acid, 15 mM NaCl, pH 7.5), 20 mM MgCl2 and 0.3% Tween 20 in PBS] for 1 h at RT, incubated overnight in primary antibodies. Secondary antibodies were applied and samples processed as described above.

The following antibodies were used: anti-Npy (1:1000; Peninsula Labs, T4068.0500), anti-Tyro3 (1:1000; Abcam, ab79778) and mouse anti-MAP2 (1:2000; Sigma, M4403), rabbit anti-calbindin (1:1000; Chemicon, AB1778), anti-5HT (1:8000; Immunostar, 20080)

For silver nitrate enhanced immunoperoxidase staining, 20 µm sections were cut through hypothalami of P19 +/+, *anx*/*anx* and *Tyro3-GFP*; *anx*/*anx* mice for anti-Npy staining, and through cerebella of P19 and P10 *Tyro3-GFP*; *+/+* and *R7W-Tyro3-GFP*;*+/+* mice for anti-GFP staining. Immunoperoxidase staining was performed with the Vectastain kit (Vector Laboratories) and enhancement with silver nitrate was performed as described in Kitt et al. (1987). Slides were scanned using a Zeiss Axio Scan.Z1 microscope. For analyses of Npy-expressing neurons, hypothalami of P19 mice were sectioned completely and Npy+ soma from nine sections containing the arcuate nucleus, median eminence and highest number of Npy+ neurons for each of three animals per genotype were counted. In wild-type mice Npy+ soma were identified by the presence of circular or semi-circular Npy staining in the soma. By contrast, Npy staining can be clearly detected throughout the soma from *anx/anx* mice and as a more pronounced circular staining within the soma of *anx/anx*; *Tyro3-GFP* transgenic animals. Thus, it is possible that the numbers of Npy+ soma is underestimated in wild-type animals, and that the difference between wild-type and *anx/anx* Npy+ neurons is even greater than recorded here. Pairwise comparisons were performed using the Wilcoxon signed-rank test (alpha<0.05; *P*<0.05) (Social Science Statistics, 2015: http://www.socscistatistics.com/tests/signedranks/Default2.aspx; accessed on 4.19.2016).

### Akt-P analysis

Tyro3-pDest47 and R7W-Tyro3-pDest47 plasmids were generated using the Gateway system (Invitrogen). Triple GFP-coding sequences in pDest47 were replaced with those of mRFP, and the Gateway system was used to generate Tyro3-RFP and R7W-Tyro3-RFP pDest47 constructs. After transfection into Neuro2A cells with Effectene transfection reagent (Qiagen), stable cell lines were generated by selecting with 500 µg/ml G418, and cell lines expressing equivalent amounts of Tyro3-RFP or R7W-Tyro3-RFP were used to assess Akt phosphorylation. Cells were serum-starved overnight, activated with serum for 15 min, harvested, lysed in RIPA buffer [1% NP-40, 0.5% sodium deoxycholate, 0.1% SDS, protease inhibitor cocktail (Roche) in PBS], incubated on ice for 10 min, and centrifuged at 4°C for 10 min. SDS-PAGE was carried out on 10% gels and transferred to nitrocellulose membranes. After blocking (5% non-fat milk, 3% BSA, PBST) at RT for 1 h, membranes were incubated with either anti-RFP (1:1000; Chemicon, AB3216), anti-Akt (1:1000; Cell Signaling Technologies, 4691) and anti-phospho-Akt (1:1000; Cell Signaling Technologies, 4060) in 5% BSA TBST at 4°C overnight, and immunoreactive antibodies were detected using the enhanced chemiluminescence (ECL) system (Perkin Elmer). Image J (NIH) was used to measure densitometry and subsequent statistical analysis was completed with GraphPad Prism software. A minimum of three separate cell lines expressing Tyro3-RFP or R7W-Tyro3-RFP were used and assays were performed at a minimum in duplicate. Data were analyzed using an unpaired *t*-test. Ratios of Akt-P/Akt were 9.7±2.3% for Neuro2A (*P*=0.015), 29.9±7.0% for T3-RFP (*P*=0.451), and 37.1±6.1% for R7W-RFP (*P*=0.451).

Cerebellar lysates were generated by homogenizing P19 *anx/+* and *anx/anx* cerebella in RIPA buffer at 4°C, incubated on ice for 10 min and centrifuging at 10,000 ***g*** for 10 min at 4°C. 10 µg of protein was analyzed by immunoblot for Akt and phospho-Akt levels. Ratios of Akt-P/Akt were 119±16% for *anx/+* (*n*=6) and 106±30% for *anx/anx* (*n*=4; *P*=0.674, nonparametric *t*-test).

### Co-translational processing

*In vitro* transcription/translation (TnT) with a Tyro3 and R7W-Tyro3 template containing all eight predicted N-glycosylation sites coupled with the addition of canine pancreatic microsomal membranes (CMM) and subsequent addition of endoglycosidase H was used to examine the cotranslational processing of R7W-Tyro3. Cell-free expression of Tyro3 and R7W-Tyro3 protein was performed using a TnT Coupled Reticulocyte Lysate System (Promega, Madison, WI, USA). DNA templates of Tyro3 and R7W-Tyro3 protein containing primary sequence for the first 410 amino acids of the extracellular domain, and, thus, all eight predicted N-glycosylation sites, were generated by digestion with *Nhe*I. TnT was performed in the presence and absence of Canine Pancreatic Microsomal Membranes (Promega) according to the manufacturer's instructions. To remove any N-acetylglucosamine residues, some products were denatured in denaturing buffer (0.5% SDS, 5 mM 2-mercaptoethanol) at 37°C for 15 min and subsequently digested with 500 U of endoglycosidase H (New England Biolabs, Beverly, MA, USA) at 37°C for 1 h. Samples were analyzed by 15% SDS-PAGE and autoradiography. β-lactamase from *Escherichia coli* was used to assay for signal peptidase activity, and α-factor from *Saccharomyces cerevisiae* to assay for core glycosylation activity.

### Neuronal cultures

Primary hippocampal and cortical neurons were harvested from embryonic day-(E)17 mice and cultured for 10-14 days as described in (Yudowski et al., 2006) and (Boucard et al., 2005). For tranfection, dissociated neurons were transfected prior to plating with the Amaxa Nucleofector kit (VPG-1001, Lonza) using approximately 5 X 104 cells and 10 μg plasmid DNA per transfection.
